# A Case of Inversio Uteri

**Published:** 1853-11

**Authors:** Sanford B. Hunt


BUFFALO MEDICAL JOURNAL

AND

MONTHLY REVIEW.

VOL. 9. NOVEMBER, 1853. NO. 6.

### ORIGINAL COMMUNICATIONS.

### ART. I.—*A Case of Inversio Uteri.* With an analysis of sixty-seven cases, collated from various sources. By SANFORD B. HUNT, M. D.

Mrs. Ann W——, of Irish birth, aged 25 years, was confined with her second child, Sept. 7th, 1853. She was attended by a German midwife, from whom, and from the other attendants, I obtained the following history: The labor commenced yesterday, she having just completed the seventh month of pregnancy. Pains continued during the night and day, until a little after 4, P. M., of the 7th, when, after a good deal of severe pain, the child was expelled. An unusually large quantity of water came away with the child. She was, at the moment of delivery, seated nearly erect in her sister's lap, and the expulsive pain which completed the labor, forced the child down suddenly, so that it dragged heavily upon the cord by its entire weight, and the uterus was instantly, and completely, inverted, and protruded beyond the vulva, lying as a large globular mass between her thighs. She was very faint, and they thought her about to die. The midwife tied the cord and removed the child. She then replaced the uterus within the vulva, and having placed her patient upon the bed, sent for a physician. The case then came under my care.

I arrived at the house about 5 o'clock, say three-fourths of an hour after the accident. She was pale, pulseless, and sighing—apparently moribund.

I ordered brandy to be given, and examined immediately. The placenta—the cord being unusually short—I found presenting at the vulva. I should estimate the length of the cord at from eight to ten inches, though it was not measured. Within the vagina was a large globular tumor, as large as the head of a child, but not to be mistaken for it. Its surface was rough, moderately firm, and bleeding. This was the uterus inverted, with the placenta firmly adherent to it, in a space of about three by four inches. At this time Mrs. W. lay in a pool of blood, and much blood was constantly escaping from her. Without much difficulty I separated the placenta, with the result of a partial check to the hemorrhage. I then made an effort to reduce the tumor by placing my hand upon the fundus, and pressing it upward, but there being considerable resistance, and as the hemorrhage, though now not great, had become (from the amount previously lost) the more immediate danger, I desisted for the moment from attempts at reduction, and dispatched a messenger for my colleague, Dr. Flint. In the mean time I applied a tampon to the bleeding surface, and cold to the pubes. These means being insufficient, I compressed the abdominal aorta against the vertebral column. The hemorrhage then ceased; and by the continual administration of large draughts of brandy, her pulse rallied, and she was in a more promising situation when Dr. Flint arrived, which was in about twenty minutes after my own advent—the manipulations described not having occupied over five minutes.

Dr. Flint removed the tampon for the purpose of an examination. He confirmed my diagnosis, and we proceeded immediately to the reduction. The open and fully dilated os uteri could be felt through the abdominal parietes over the pubis. Dr. Flint engaging his fingers behind this externally, I introduced my entire hand into the vagina, and gradually carried the tumor up. The fundus, at first with considerable opposition, slowly yielded and rolled in upon itself, until the usual prominent tumor over the pubis being felt, Dr. Flint thought the reduction completed. To convince him to the contrary, I withdrew my hand, and Dr. F. inserting his own, found the posterior wall of the uterus still mostly engaged in the os. By gentle manipulation he soon restored this to its normal position, and retaining his hand within the uterine cavity until contraction came on, the reduction was completed, quietly and safely. On again examining, I found the os uteri *in situ*, and well contracted. Dr. Flint leaving soon after, I gave brandy *pro re nata*. Our patient fell asleep immediately after the reduction. She was very faint and prostrated for some time. At 8, P. M., I bandaged her, and applied cold to check flowing, which was then renewed.

℞ Morph. sulph., gr. ¼, every 4 hours.

*September 8th.*—I found a very smart reaction. She had headache, her pulse 120, full and bounding, but soft. Moderate flowing. Urinates with ease.

℞ Sulph. morph., gr. ¼, every 4 hours.

*September 10th.*—Up to this time I have maintained the morphia as above. To-day there is febrile disturbance, and abdominal tenderness. Breasts swollen; milk profuse. Attributing this febrile action to the ordinary milk fever, I gave a saline cathartic. Aside from subsequent difficulty in procuring sufficient action of the bowels, Mrs. W. went on to convalescence with no very peculiar symptoms. She now, (*Sept. 30th,*) sits up most of the time, and is in a favorable condition.

Since the occurrence of this alarming and nearly fatal case, I have been led from a consideration of the imperfect state of information on this branch of obstetric science, from the differences of opinion as to its proper management, and from the great importance, both as to the life of the patient, and the reputation of the practitioner, of a clear and definite idea of its real nature, to collate from such sources as were within my reach all cases of which I could find any record, and to furnish the results of this labor to the readers of the Journal in a condensed form.

Of many of these cases I found the records sufficiently full to enable me to ascertain their most important phenomena. In other cases the reports were so meagre, that I could only ascertain a very few of the symptoms present. I have endeavored to present them in such a form as would give to the reader a fair and uncolored view of the facts, and in every case where the occurrence of a symptom was doubtful, I have omitted to mention it.

I shall first give as brief a summary as possible of the results of my analysis, and subsequently shall draw from them such inferences as to treatment as they seem to teach. I trust that these deductions may possess some value. No one man is able to speak authoritatively from his own experience on this subject. So rare is the accident, that the most extensive practitioners frequently pass a lifetime without meeting a single case, while it has often happened, that upon the man of limited resources, and narrow obstetrical experience, has fallen the responsibility of treating this dislocation.

The total number of cases of which I find on record, is *sixty-seven:*
Of these, *twenty-one* were reduced and recovered;
*Thirty-three* became chronic; and

*Thirteen* died.

The number of the labor is mentioned in *twenty-three* cases:

*Nine* occurred in the first labor;

*Seven* in the second labor;

*Six* are mentioned as multiparous; and

*One* occurred after abortion, at the fourth month.

The degree of inversion is mentioned in *forty-one* cases:

*Thirty-five* were cases of complete inversion; and

*Six* were mentioned as partially inverted.

The time at which the accident occurred is noticed in *forty-five* cases:

*Nineteen* of these happened immediately on the delivery of the child;

*Eight* on attempting to remove the placenta.

The remaining *eighteen* cases occurred:

*One*, after removing the placenta by the hand in the uterus;

*One*, simultaneously with the first after-pain, seventeen minutes after the expulsion of the child;

*Three*, half an hour after the delivery of the child;

*One*, eighteen hours after labor;

*Three*, three days after parturition;

*One* on the eighth day;

*One* on the twelfth day;

*One* on the fifteenth day;

*One* on the tenth day;

*Four* are mentioned as occurring at unknown periods; and

*One*, nine days after abortion at the fourth month.

The condition of the placenta is mentioned in *thirty-seven* cases:

In *eighteen* of these it is mentioned as adherent.

In *nineteen* it was removed either before the occurrence of inversion, or without recognizing the difficulty.

In *ten* cases the placenta was intentionally detached when adherent.

The records as to hemorrhage are very imperfect.

In only *six* cases is the degree of hemorrhage *before* removing an adherent placenta mentioned:

In *one* of these it was slight. This case was fatal.

In *five* it was large in amount. One of these was fatal.

The degree of hemorrhage *after* detaching an adherent placenta, is mentioned in *ten* cases:

In *eight* of these it is mentioned as slight.

In *two* it was dangerous. One of these was fatal.

Hemorrhage is noticed after removing the placenta as usual, or before the accident, in *five* cases.

In *eight* cases it is spoken of in general terms.

Of *two* cases where the placenta was returned with the uterus in the operation of reposition:

*One* had fatal hemorrhage; while in the *other* it was slight.

In *five* cases no hemorrhage occurred.

Convulsions occurred in *three* cases; all of which were fatal.

Syncope in *twelve* cases.

Syncope, without hemorrhage, in *one* case.

The above closes the record of the more important symptoms occurring in the recorded cases. It is to be regretted that it is not more complete, but a careful revision has not enabled me to add anything to them. In the consideration of the phenomena of fatal and irreducible cases, other facts of importance will be deduced. I shall first, however, attempt a review of the causes of this accident, and shall, as far as the facts in my possession enable me, endeavor to show the more usual causes of the dislocation.

*Ten* cases were attended by female midwives.

In *ten* cases there was undue traction on the funis.*

In *nine* cases there was a rapid labor.

In *five* cases the patient was delivered in the erect posture.

In *five* cases there was a short funis.

In *one* case it was perhaps attributable to sneezing, produced by snuff, given to accelerate the delivery of the placenta.

In *one* case it occurred on getting out of bed, on the third day.

In *one* case on leaving the bed, on the fifteenth day.

In *one* while at stool, on the tenth day; and

In *one* there was placenta prævia.

Of the circumstances bearing upon the treatment of the accident, the record is more full and satisfactory. I have already enumerated *twenty-one* cases as recovering after reposition.

In *four* other cases death occurred after the reposition of the organ.

Of the irreducible cases *thirty-three* became chronic, and *five* others died

---

* Dr. Wm. Hunter estimated the circumstances under which traction on the funis may produce inversion very correctly, when he said that, "When a uterus is flaccid, it is inverted as easily as the finger of a glove; but when it is hard, globular, and contracted, it is as difficult to invert as a jackboot."

from consecutive circumstances. I defer the details until I speak of the treatment.

Of the *thirteen* fatal cases it is recorded that death occurred immediately and before the reposition of the organ could be effected, in *four* cases.

In *four* other cases, death occurred after the reposition of the uterus, and the remaining *five* deaths occurred in chronic cases.

*Remarks.* The twenty-three cases in which the number of the labor is mentioned, would indicate that the tendency to inversion decreases with the number of children which the woman has borne.

| | | | |
|---|---|---|---|
| The proportion of first labors | | | is 1 in 2·55. |
| " | " | second " | is 1 in 3·28. |
| " | " | multiparous labors | is 1 in 5·83. |

*Causes.* The cause to which the inversion is attributed, varies very much in different cases. It is noticed that 1 in 6·7, were attended by female accoucheurs. As the proportion of labors occurring under the supervision of female attendants is undoubtedly much less than 1 in 6·7, it is probable that a lack of necessary skill and precaution were influential in producing the accident. But it is by no means fair to draw the inference that a want of skill is always, or even generally the cause. Dr. Meigs details a case, (the one mentioned as a placenta prævia,) in which, after the delivery, the fundus came down, was easily replaced, but again came down, and was speedily fatal. It will also be noticed, that nineteen of sixty-seven cases occurred immediately after the delivery of the child. In these cases it is difficult to suppose that any cause within the control of the attendant could have operated, except the erect position, which was mentioned in *five* cases. A short funis is another cause which is probably the most frequent of any, though only mentioned in five cases. The erect posture, combined with a short funis, would almost inevitably produce inversion. It is highly probable that the funis is the more frequent cause, from the fact that so large a portion of the cases had adherent placenta—1 in 3·53 of the entire number of cases are mentioned as adherent. As there is nothing in the adhesion itself to bring on inversion, we may suppose that it furnishes the condition necessary to make a traction on the cord, either accidental or intentional, the source of the dislocation. In 1 in 6·7 of the cases undue traction was made upon the cord. It is now the habit of many excellent and careful accoucheurs to deliver the placenta immediately. Without the usual and proper precaution of placing one hand upon the fundus of the uterus while making traction with the other, it is plain that the practice alluded to would be mischievous. With this precaution there can be no danger in prudent hands. Rapid labor is mentioned in nine cases. No mention is made in the records, of the quantity of amniotic fluid. In my own case there was an unusually large discharge of the waters occurring at the moment of delivery. The other causes enumerated are evidently exceptional. One inversion occurred on getting out of bed on the third day; one from the same cause on the fifteenth day, and one at stool on the tenth day.

*Degree of Inversion.* Only two divisions have been made in this analysis,—partial, and complete inversion. Another division, or rather stage of inversion, has been made by some writers—that of depression. It is supposed that in those cases of inversion occurring at a length of time after placental delivery, a slight depression exists in the fundus, from the time of removing the placenta; that an inverted action of the uterine fibre being thus produced, it goes on gradually increasing the depression with each after-pain, until it at last becomes partial, or complete. It is not improbable that this condition may sometimes obtain. The treatment proper for this stage would be the introduction of some firm instrument, like a rectum bougie, into the cavity of the uterus, and restoring the fundus to its natural shape by pressure from within. The operation would be a simple and easy one, but its necessity could not, as a general thing, be detected in time to make it useful.

By partial inversion is intended that condition when the fundus protruding through the os tincæ, the inversion is still incomplete, the os itself forming a ligating band about the substance of the organ. Of the *forty-one* cases in which the degree of inversion is noted, only *six* are mentioned as partial. It is probable that but few cases remain long partially inverted; they going on either to complete inversion, or to spontaneous reposition. That the latter is not impossible, is proved by a number of cases to be hereafter enumerated, and the argument to be drawn therefrom is, that it would not be judicious to do as recommended and practiced in one instance by a distinguished American accoucheur, who, in a case of partial inversion, dragged down the fundus, and made it a complete inversion, in order to prevent ligation by the os.

Complete inversion is mentioned in *thirty-five* cases. It is thus evident either that the complete is by far the more common form of the accident, or that the partial form is not so often detected; a consideration of some probability, and likely to result unfortunately, for it does not appear that partial inversion is any more manageable in its chronic stage than is complete; and the consequences of the ligation by the os may be much more serious, producing much and obstinate hemorrhage, and, not unfrequently, gangrene of the excluded portion. It is probable that in all cases of complete inversion the uterus is at first protruded through the vulva. The displaced organ can, however, without difficulty, be returned within the vagina. In cases of complete inversion, the peritoneal surface of the displaced organ forms a cavity, in which are contained the ovaries and fallopian tubes, and sometimes portions of the intestine. The internal, or mucous surface is turned outward as we turn a glove. It formes a globular tumor, of a size proportioned to its recent character. It has a rough rugose surface, firm to the touch, and bleeding when handled.

*Treatment.* In one-half of the cases of inversion, the practitioner will find the placenta attached to the inner, and now conical, surface of the uterus. Should the placenta have been already removed or expelled, there is no doubt as to the proper course to be pursued. But when the placenta still remains adherent, there is room for discussion as to whether to replace the uterus with the placenta still attached, or to first detach it, and then reduce. The weight of authority is in favor of the former method of procedure. The argument adduced in its favor, is the danger of increased hemorrhage attendant on the removal of the after-birth. In support of this argument are arrayed many of the most distinguished names in obstetrical science.

Burns says: "If the placenta still adhere, we should not remove it till we have reduced the uterus; after which we excite the contraction of the womb to make it throw it off."

Denman, after advising the removal of the placenta when only partially adherent, adds: "But if the placenta should wholly adhere, it would be better to replace the uterus before we endeavor to separate the placenta. The ground of this opinion is, that while we are separating the placenta the cervix of the uterus is contracting, and the difficulty of replacing it is increasing, which is a greater evil by far than a retained placenta."

Dewees (System of Midwifery, p. 466): "If the placenta offers itself before the prolapsed fundus, we may, if detached, deliver it immediately; but if it be adherent, and the mouth of the uterus does not offer too much resistance, it must be carried up with the fundus, and separated as before directed."

Gooch (Midwifery, p. 273): "First make an attempt to replace the uterus without separating the placenta from it; and if you succeed so much the better; then by external pressure and friction, excite the action of the uterus to separate and expel the placenta."

Besides the authors above quoted, Clarke, Carus, Newnham, and Blundell, record a decided protest against removing the placenta prior to the reduction of the displaced organ. All of them, however, admit that their method renders reduction more difficult, and in cases where the bulk of the placenta is such as to render its return impossible, they permit its removal.

On the other side of the question we find Bandelocque, Boivin, Churchill, and Moreau. As my own opinion coincides with those last mentioned, I shall briefly state the reasons which govern my decision, and the facts from my analysis which bear upon it.

It is hardly probable that the placenta will in any case of inversion be found attached in its whole surface. The turning outward of the uterine cavity must necessarily detach it in some portion of its surface.

In *nineteen* out of thirty-seven cases in which the condition of the placenta is recorded, it was detached without recognizing the difficulty, or expelled spontaneously. It is not to be supposed that these placentæ were adherent.

In the *eighteen* remaining cases the placenta was adherent. In only *six* of these is it recorded that any attempt was made to return the placenta. *Two* of these attempts failed; in the other *four* the placenta was carried up with the uterus. In *one* of these four cases, there was fatal hemorrhage after the reduction. In another, the placenta remained attached for four hours after reduction. In the *third* case, the placenta was not removed till five days after reduction. Of the *fourth* and last case, no record is made of subsequent difficulties.

It would seem from these facts, that whatever may be the danger of immediately detaching the secundines, their return is also a formidable operation, attended by great difficulty in performance, and when successful, leaving another and dangerous condition still present. Some of the facts in our analysis will go to show that the danger of hemorrhage has been greatly overrated.

In the first place, out of the thirteen deaths, only *four* died from hemorrhage.

Of these *four*, *one* died after the reduction of the placenta with the uterus.

*One* died *prior* to detaching an adherent placenta.

*One* died, the placenta being expelled previously to recognizing the accident.

*One* died from hemorrhage consecutive to removing an attached placenta.

Thus we have only *one* death in sixty-seven cases, which can be attributed to detaching the placenta. The history of other fatal cases will—incidentally—farther illustrate this point in treatment.

The arguments thus adduced are:

*First;* the attempt to return the placenta very much increases the difficulty of reposition.

*Second;* when successful it leaves a formidable difficulty to be still encountered.

*Third;* it does not decrease the danger of hemorrhage.

By removing the placenta immediately you avoid these difficulties without increasing the danger.

The placenta having then been removed, our next step is to replace the uterus. But here another difficulty, not mentioned by any author, may intervene. The hemorrhage may have been so great as to be of more immediate consequence than the displacement itself. There may be after-pains present in the uterus, offering a resistance to manipulation too great to be overcome. In my own case, both these happened. On removing the placenta I attempted immediate reposition, but the resistance of the after-pain was such as to render it impossible. The flooding, too, though much less than before the removal of the placenta, was still, in her exhausted condition, very formidable. I, therefore, during the delay previous to Dr. Flint's arrival, directed my efforts to checking the hemorrhage, and restoring consciousness. This accomplished, the patient was in a much more favorable condition for reduction than a few minutes previously.

The hand is the best instrument for reduction. It is manageable and always present. Some writers have recommended the head of a walking-stick, a large bougie, or any other blunt, rounded instrument. Such an one would be liable to slip from the globular surface, and do injury to the parts, and has no advantages to compensate for this danger. Others have proposed that the hand itself should be shielded by a cloth—why, I do not know. First returning the uterus within the vagina, it should be carried bodily up until the vagina is "on the stretch." At this point it has been advised by Mr. Newnham to "return first that portion of the organ which was last excluded from the os." How this is to be done in *complete* inversion does not appear; in *partial* inversion it is possible, and is, I opine, the usual method of completing all reductions. When the fundus is returned within the os, the womb passes in its progress through all the degrees of partial inversion; and it is then convenient to pass the fingers above the everted lips of the os, and to press the excluded portion upward with the thumb. In all cases, the whole hand should be introduced. The pressure should be firm, and continued for some time, even if the os does not yield at first. Dr. Dewees has said that he does not believe it possible to return a complete inversion, though he adds that he cannot speak from positive knowledge. A complete inversion may be as readily returned as a partial one, provided there is not too great contraction of the os. Now complete inversion does not necessarily suppose such contraction. The os may be dilated to its fullest extent. The fact of inversion predicates a lax fibre, and very many cases of unmistakably complete inversion are recorded as reduced. The uterus having been restored to its natural position, the hand should be maintained within its cavity until contraction takes place. The after treatment does not require consideration here. Only one fact need be mentioned: I have found no case recorded where the patient died from subsequent disease, such as metritis, or peritonitis.

I have already enumerated a portion of the fatal cases—those which had any bearing on the question of removal of the placenta.

Death occurred immediately, and before reposition, in *four* cases.

*One* died from hemorrhage before the difficulty was detected. The placenta had been removed.

*One* from hemorrhage from an attached placenta, which was not removed.

*One* from hemorrhage subsequent to the removal of an adherent placenta.

*One* from "nervous exhaustion with slight hemorrhage."

*Four* deaths occurred immediately after reduction. *One* from hemorrhage, the placenta having been returned with the uterus; and *three* from convulsion. The remaining *five* of the thirteen deaths were in chronic cases. We have thus considered the principal features of the accident. Only one consideration remains—that of the period after inversion at which reposition may be effected. As time passes on, the uterus contracts, its parietes become thicker and firmer, and the os uteri is more closely contracted, and less readily dilated to admit the return of the excluded portion. Every consideration combines to make an immediate reposition desirable and necessary. The delay of even a few moments is to be regretted, while the passing of an hour may forever forbid replacement. An hour after the accident is considered a long time, and likely to make the operation unsuccessful. Dr. Locock speaks of having succeeded at the end of an hour and a half, in a tone which indicates a pleasurable disappointment. But lest the practitioner should be too readily discouraged, many cases of successful attempts at reduction are recorded at much longer periods. In recording these cases, I have, in most instances, preceded the length of time at which the reduction was effected by the name of the accoucheur. This I have done, that the character of the reporter may in some measure settle the credibility of the report. Dr. Ayer, of Boston, thirty-one hours; Dr. Merriman, "long afterward;" Dr. McCoy, 2 days; unknown reporters, 12 hours, 57 hours, 4 weeks. Besides these are the following cases from Churchill, which are not elsewhere included in this analysis: Suffler, 6 or 7 hours; White, 17 hours; Wynter, 24 hours; Dickenson, 27 hours; Cawley, 3 days; Dr. Radford, 7 days; Chopart and Ane, 8 days; Ingleby,.8 days; Lanverjat, 10 or 12 days; Hoin, 13 days; Dr. Belcombe, 12 weeks.

Churchill evidently considers these cases authentic. They are nevertheless exceptional cases, and should not be admitted in excuse for any delay of immediate reposition.

That the consideration of *chronic* inversion is not less important than that of the immediate accident, is evident from the fact, that of the sixty-seven cases enumerated, *thirty-two* were either irreducible or not reduced, and became chronic. The term chronic, though not strictly applicable, is still sufficiently descriptive. It means that condition which is expressed in the word irreducible. I have no sufficient data for explaining *why* so large a proportion of the cases were not reduced. In many instances it is evident that the accident was not detected until so long a time had elapsed that reduction was impossible. In a very few it is stated by the reporters, that though the accident was discovered early, the condition of the organ, and especially of the os uteri, was such as to preclude reposition. We have already quoted the opinion of Dr. Dewees that a complete inversion is necessarily irreducible, and though we think it sufficiently shown that this is not even frequently the case, yet it is easy to conceive that there might be such a tonic contraction or rigidity of the os, as to preclude any possibility of dilating it sufficiently to replace the organ. In such a condition it is evident that no safe amount of force would result in success. I find reason to believe, from the different reports I have read, that most cases of the chronic form were insidious in their oncoming. The pain resulting from a simple depression of the fundus might easily be mistaken for after-pain of unusual severity; and this depression may go on through the various stages of partial, until at last it becomes a complete inversion. Or, supposing the displacement to be only partial, the true state of things would be very liable to escape detection until too late for any remedial measures.

Chronic inversion is not necessarily complete—it may be partial. To this latter form of the disorder I shall first direct my inquiries. A partial inversion would not protrude beyond the vulva. The physical signs of its presence would be a depression in, or entire want of the usual globular tumor over the pubis; while an examination, per vaginam, would reveal there a globular mass, protruding from the os uteri, and more or less ligated by it. Our attention should be directed to the possibility of inversion, by the presence of unusually severe uterine pain, of hemorrhage, of syncope or convulsion, and of retention of urine. The sensations of the patient would be the same as those attendant on prolapsus uteri, but in a more aggravated form. If to these sensations we add the occurrence of syncope and hemorrhage, or of syncope without hemorrhage, we should have sufficient reason to suspect inversion.

It has been remarked that partial inversion was, in some sense, a more important and dangerous accident than complete. From the greater degree of ligation by the os, there would be greater danger of hemorrhage, of strangulation, and of consequent gangrene. The hemorrhage, too, would be more uncontrollable. Added to this, the accident is less liable to detection, and is not in all cases more readily reduced than is the other form.

Relative to the treatment of partial inversion, there can be no great difference between it and that proper for the complete form. The greater liability to gangrene, however, makes a distinction which should not be overlooked. I find *five* cases in which gangrene occurred. Singularly enough, all of these resulted in what may be called a cure; that is, the cases recovered from this amputation by natural process, and regained a comfortable general health. I do not find any cases which were fatal from gangrene. An inference which may be drawn from this fact would go to prove that the proposition of Dr. Dewees, (and which in one instance he carried out,) to pull down upon an irreducible partial inversion, and render it complete, is not justified by the ulterior consequences of gangrene. It is to avoid gangrene that Dr. Dewees proposes this plan, but until a greater amount of evidence is furnished of the fatality of gangrene, the operation can hardly be justified. It should not be forgotten, however, that very many complete cases enjoy a comfortable degree of general health. Were it proven that the probability of regaining such a degree of health under complete inversion, is greater than the chance of a cure by gangrene, then the operation would be worthy of consideration. As yet, no sufficient data exist to decide this point.

But no case of partial inversion should be readily surrendered to either of these uncertain chances. Every attempt at reduction should be made. Methodical compression of the protruded portion should be employed, and all such local applications as would be likely to relax the constricted fibres of the os, and diminish the size of the vaginal tumor. Among these I would suggest, as most likely to be beneficial, the application of belladonna to the os.

The hemorrhage should be controlled by the local use of astringents, and very firm and long continued pressure should be made.

The propriety of surgical interference will be considered under the head of complete irreducible inversion; and all other remarks as to treatment will apply to either form of the accident.

I find (aside from the five cases of cure by gangrene) that of the irreducible cases, *five*, or nearly one-sixth, terminated by *spontaneous reposition*. For a long time the possibility of this very fortunate occurrence was denied by authors, but the occurrence of two cases in France, and subsequently of three in this country, have placed the matter beyond a doubt. Few incidents in the self-instituted processes of nature for the cure of the disease are more interesting than this. Only the authentic sources by which the cases are furnished could convince us of the possibility of so great an effort to accomplish a restoration, not of function only, but of natural position, against so many chances of failure. I trust I shall not be tedious if I devote some space to the history of these cases.

The first was that of Madame De La Barre, the wife of a surgeon of Beuzeville. Six months after the occurrence of inversion, this lady, in getting out of bed, fell upon the floor. At that moment she felt an extraordinary motion in her belly, accompanied by very severe uterine pain, which was followed by syncope. On recovering from this condition, it was ascertained that the uterus had returned to its natural position.

The second case is the famous one of Madame Boncharlatt, authenticated by Baudelocque. Madame B., after suffering for seven or eight years with inversion, consulted Baudelocque, who failed in an attempt to reduce it. On the evening preceding the day on which another attempt was to be made, her friends insisted upon her walking about the room. She struggled and fell upon the carpet, and immediately felt an unusual movement and pain in the uterus, followed by momentary unconsciousness. Baudelocque being called, found that spontaneous reposition had taken place. This woman, having recovered her health, subsequently gave birth to a child.

To come down to more recent times. In the Boston Medical and Surgical Journal, vol. XL, p. 277, Dr. I. C. Hatch, of Kent, Mass., communicates the case of Mrs. H., who had inverted uterus, the diagnosis being confirmed by Prof. Beers, of New Haven, who made an attempt at reposition. At the end of nine or ten months, Mrs. H. found that the tumor in the vagina had so changed its place "that she did not know what had become of it." She afterward bore a child at full time.

Dr. Meigs also relates two cases of gradual and spontaneous reposition: one, after three years had elapsed; the other, after many months. Both these cases subsequently bore children.

We have thus disposed of *ten* of the thirty-two irreducible cases by spontaneous cure—*five* of them by gangrene, and consequent mutilation of the organ, and *five* by what we can only consider as a most remarkable accident—the spontaneous reposition of the organ, and its restoration to integrity of function, as proved in four cases by the occurrence of subsequent pregnancy. It may not be amiss to mention, in this connection, another case of remarkable interest, if there is no error in the diagnosis, or hiatus in the history. This case involves the possibility of conception taking place in an inverted uterus. The wife of Julian Roussin gave birth, in October, 1777, to a healthy child, but the midwife, in extracting the placenta, inverted the uterus. The tumor was merely returned into the vagina. Ten months afterward, she suspected herself pregnant. At three months, after a good deal of pain, she expelled a considerable mass, which MM. Thuilier and Vager recognized as an inverted uterus. For several days M. Vager tried to reduce it, but could not, and at last, by advice of MM. Thuilier and Paroifreu, he returned it to the vagina. Six days after, Madame Roussin expelled a fetus five inches in length. The woman was subsequently examined by M. Chevruel, who found the uterus still inverted. He supposed it a case of fallopian pregnancy. Probably such a case will never occur again, and there is much room to doubt the history of this case. No one but a midwife saw it till after the abortion. By supposing the original accident to have been prolapsus, the inversion to have occurred at the moment of abortion, the fetus to have remained in the vagina while the uterus pushed on through the vulva, and finally, the fetus to have been expelled by some casual movement after six days, and we have done away with all the mystery. Such is the explanation of this history offered by M. Moreau.

Two other terminations of inversion remain to be considered, first, using only such means as may tend to allay the inconveniences and dangers attendant on the condition; and second, the cure by operation.

Upon the measure of health and comfort attainable in the first mode of management, must depend, in some degree, the propriety of the operation. In a case related to me by Prof. James P. White, of this city, to which he was called some twelve or fourteen days after inversion had occurred, which was then for the first time detected, and had become irreducible, he resorted to such a treatment as it seems to me is most appropriate to such cases. The hemorrhage and discharges were controlled by astringent injections; the uterus was replaced as high up as possible, and maintained there by a pessary made of curled hair, inclosed in oil silk. The recumbent position was maintained until the parts could accustom themselves to this new condition; and finally, there was a restoration to a very comfortable degree of health, so that the patient was able to attend to her ordinary household duties, and even to ride, occasionally, a distance of some miles, to this city. That such may be the result under judicious treatment, is evident from the whole history of chronic inversion. At the moment of the accident, the womb is in a condition of hypertrophy. Its enormous size can scarcely be contained within the vagina, and accordingly we find it generally extruded from the os externum, and lying between the thighs. At this time it is subject to frequent hemorrhage. The patulous orifices of the placental vessels are still inclosed, and at the slightest handling, pour out blood profusely. But after the uterus is returned to the vagina, and is thus shielded from the air, and from irritating contact with surrounding substances, the ordinary and normal diminution of the uterus after parturition takes place; the lochial discharge is instituted, and finally, the organ regains its natural unimpregnated size, and performs its menstrual function uninterruptedly. Dr. White tells me that he has seen the menstrual secretion oozing *guttatim* from the mucous surface. The data of irreducible cases are insufficient to say what proportion of them may result thus favorably. In a case read before the New York Medical Association, by Dr. Van Pelt, the uterus was, (owing to circumstances unnecessary to be mentioned here,) found on the morning of the fourth day after inversion, lying externally to the vulva, and so concealed in dry, hard clots, which had formed it, that Dr. V. P. could not decide on the nature of the accident until they had been softened by fomentations, and removed. This uterus was reduced. This case is quoted as showing the little danger of metritis which exists, and the gravity of accident which may be recovered from. In other cases, (two in number,) the patients are mentioned as living in comfortable health some years after inversion.

These facts and inferences have all a bearing upon the question of operation for the removal of the uterus. If the patient enjoys any comfortable degree of health, there should be no operation. The only circumstances under which the knife or ligature are justifiable, are those involving great danger to the patient's life. A case of inversion in which the organ had diminished to nearly its natural size, could present symptoms no worse than those of ordinary prolapsus. We believe that the knife has not yet been proposed for this latter displacement. The circumstances which militate against an operation are briefly these:

1st. The probability of the system's accustoming itself to the accident, and regaining a degree of health.

2d. The probability of spontaneous reposition; and

3d. The dangers attendant on an operation.

The data of this analysis which bear on the propriety of operation are as follows:

There were *thirty-two* irreducible cases. Of these,

*Five* terminated by spontaneous reposition;

*Five* by strangulation and gangrene, followed by recovery;

*Four* are recorded as successful cases of *ligation;*

*Six* are mentioned as "extirpated" successfully; and

*Four* cases of ligation were fatal.

It will be seen that *one in three and a half* of the cases operated upon, died. Of the *eight* cases remaining, there is no record except of the *two* already quoted as in comfortable health years after the accident. I shall quote somewhat largely from the histories of these operations.

Of the *four* successful cases of ligation, *two* were tied, supposing them to be polypi. These, of course, are to be considered merely as fortunate terminations of unfortunate blunders.

The third of these cases is reported by Dr. Usher Parsons, of Rhode Island, in the Boston Medical and Surgical Journal, vol. xlv, p. 511. It was a partial inversion of four years' standing. There had been great suffering from alarming hemorrhage, leucorrhœa, nervousness, and uterine pain. The tumor was ligated just below the point of stricture, at the os tincæ. Four days after, the operation was completed by the knife and scissors. It resulted in a cure.

Of the fourth and last case of successful ligation, I have no record beyond the mere fact of the operation.

Of the *six* successful cases of "extirpation," it is not stated whether the knife or ligature was used. One of these was extirpated on the fourteenth day after labor, by an English surgeon — a most unjustifiable and ignorant proceeding.

Of the *four* cases of unsuccessful ligation, *one* was ligated immediately after delivery, the uterus being mistaken for a tumor in which the placenta was implanted. On the eighteenth day after, she was admitted into Boyer's ward at La Charité. Seven days after, the ligated portion came away, and she died a few days later.

*Another* case was also ligated by mistake, six months after inversion. She died five days after the operation. The *two* remaining cases died of peritonitis occurring after the operation.

Thus, of *fourteen* cases in which surgical interference took place, *ten* recovered and *four* died. Of the *four* who died, the operation was performed in ignorance of the true condition of the parts in *two* cases — a fact which would have more weight, were it not that *two* of the successful operations were also in the same category of surgical blunders.

No record of amputations of the womb would be complete without some mention of Mr. Crosse's tables, collated with especial reference to the success of this operation. It is probable that some, perhaps most, of the cases I have recorded, are also included in Mr. Crosse's statistics. A portion of them, however, are too recent, and too little known to have found place in his records.

Mr. Crosse has collected thirty-three cases of operations, from various sources.

*Nineteen* cases were treated successfully by ligature.

*Five* were treated unsuccessfully by ligature, of which *three* died.

*One* case was treated successfully by excision.

*Two* cases of excision were unsuccessful; and

*Five* cases successfully, and *one* unsuccessfully, by ligature and excision combined.

Thus the average of unsuccessful cases according to Mr. Crosse, is *one* in *four and one-tenth.* The average of my analysis is more fatal, being of fatal cases *one in three and five-tenths.* Adding the two together, we have twelve deaths in forty-seven operations. One circumstance should be mentioned: In very few of the cases, (I think in only one — that of Dr. Parsons,) is it recorded whether the inversion was partial or complete. I consider this an important point. In removing a portion only of the uterus, as in partial inversion, the danger would be much less than in the complete form. In the former case, the operation being always performed just below the point of ligation by the os, the integrity of the peritoneal cavity is not at all invaded, except at the point where the peritoneum forms the lining membrane of the abnormal inverted cavity of the uterus. The ligation by the os would effectually prevent the effusion of blood, or check the progress of inflammatory action into the peritoneal sac. But in complete inversion the converse of these propositions holds true. The peritoneal cavity is largely invaded, and there is nothing above to check effusion.

I have thus completed the consideration of this subject. It has been extended to greater length than I had at first anticipated; but the reader will readily appreciate how it is that a subject widens and extends as we investigate. Some sources of error exist in this analysis. The literature of *inversio uteri* is scattered through many books and periodical journals. Many of the records are unsatisfactory—some, perhaps, are false. I have admitted nothing as a fact that was not distinctly stated as such, while I have taken it for granted that all the records were originally written in a spirit of truth.

There is another source of error. How many cases of inversion have died unrecognized, and been recorded only as sudden and fatal floodings, convulsions, or syncopes, no one can tell. How many which have been recognized, have been concealed, or not reported, is also unknown. The impulse which we feel to report a successful case, and our repugnance to immortalize our failures, is so natural, that it not unfrequently detracts from the relative value of our statistical records. But from these records, such as they are, I have drawn the following conclusions as to the causes of inversion, its treatment, and its terminations:

The liability to inversion decreases, but not to any marked degree, with the number of children which the woman has borne.

That no one cause can be assigned as universal, but that the complication of a short funis with a rapid labor, the erect posture when delivered, and a large quantity of amniotic fluid, are the conditions most frequently present as causes.

Inversion may occur without neglect, or undue interference on the part of the accoucheur.

The placenta is adherent in a large proportion of the cases.

When adherent it should be removed prior to any attempt to reduce the inversion.

Such removal of the placenta does not increase, but rather decreases the risk of hemorrhage; while it facilitates reduction.

The returning of the placenta in reducing the inversion complicates the after treatment, and adds to the danger of hemorrhage, while it retards and renders difficult the reduction.

Hemorrhage does not occur to any more fatal extent than does convulsion or syncope.

All cases in which convulsion occurred were fatal.

There is little danger of metritis occurring after reduction.

Reduction increases in difficulty with the length of time which elapses before it is attempted. Partial inversion is less easily detected, but more readily reduced than complete.

No operation for extirpation should be resorted to until it is evident that life is endangered.

A sufficient number of cases occur in which either gradual diminution in the size of the organ, or spontaneous reposition occur, to justify and demand a delay of any operation for extirpation, until it is urgently called for by the imminent danger of the patient.

The operation by ligature, involves less danger than that by excision, and is therefore preferable to it.

An operation is more frequently necessary in partial than complete inversion, and is at the same time less dangerous.

Finally, under judicious local and general treatment, an inverted uterus may often exist for many years without great loss of locomotion or of usefulness, and with a comfortable degree of general health.

